# Supplementary material for: Clinically translatable quantitative molecular photoacoustic imaging with liposome-encapsulated ICG J-aggregates
Source: Nat Commun. 2021 Sep 13;12:5410. doi: 10.1038/s41467-021-25452-3 (PMC8438038; doi:10.1038/s41467-021-25452-3)
Supplement: Supplementary file 1 — Supplementary Information [file 41467_2021_25452_MOESM1_ESM.pdf]

Supplementary Information

Clinically translatable quantitative molecular photoacoustic imaging with liposome-encapsulated ICG J-aggregates

Cayla A. Wood[1,2], Sangheon Han[1,3], Chang Soo Kim[1], Yunfei Wen[4], Diego R.T. Sampaio[1,5], Justin T. Harris[6], Kimberly A. Homan[6], Jody L. Swain[7], Stanislav Y. Emelianov[8,9], Anil K. Sood[2,4], Jason R. Cook[6], Konstantin V. Sokolov[1,2,3,10*†], Richard R. Bouchard[1,2*†]

[1] Department of Imaging Physics, The University of Texas MD Anderson Cancer Center, Houston, TX, USA

[2] The University of Texas MD Anderson Cancer Center UTHealth Graduate School of Biomedical Sciences, Houston, TX, USA

[3] Department of Bioengineering, Rice University, Houston, TX, USA

[4] Department of Gynecologic Oncology and Reproductive Medicine, The University of Texas MD Anderson Cancer Center, Houston, TX, USA

[5] Department of Physics, Faculty of Philosophy, Science and Letters of Ribeirao Preto, University of Sao Paulo, Sao Paulo, Brazil

[6] NanoHybrids, Inc., Austin, TX, USA

[7] Department of Veterinary Medicine and Surgery, The University of Texas MD Anderson Cancer Center, Houston, TX, USA

[8] School of Electrical and Computer Engineering, Georgia Institute of Technology, Atlanta, GA, USA

[9] Wallace H. Coulter Department of Biomedical Engineering, Georgia Institute of Technology and Emory University School of Medicine, Atlanta, GA, USA

[10] Department of Biomedical Engineering, The University of Texas at Austin, Austin, TX, USA

[*] Corresponding authors: ksokolov@mdanderson.org; rrbouchard@mdanderson.org

[†] These authors contributed equally.

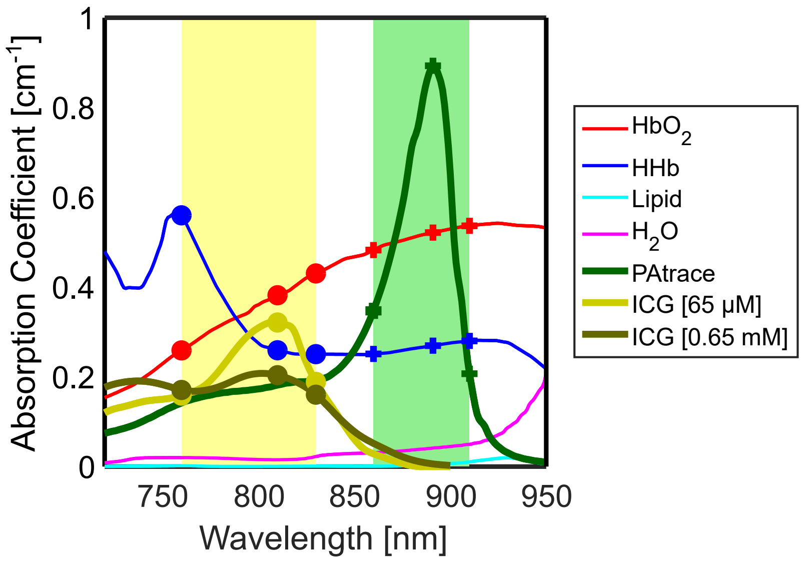

Supplementary Fig. 1: Spectral comparison of photoabsorbers. PAtrace (dark green trace) presents with sharp spectral features (lime region from 870-920 nm), where the spectra from hemoglobin (red, blue traces) remain relatively flat. This provides PAtrace with robust detection sensitivity to sample its ~890nm peak. Further, its relatively flat spectrum from 760-830 nm facilitates accurate quantification of PA-based SO_2, which is critical for understanding dynamics associated with tumor microenvironment regularization. Separation of monomeric ICG (gold/olive traces; spectra provided in blood serum) from hemoglobin, on the other hand, requires multi-wavelength imaging in a (light yellow) region that overlaps with distinct spectral features from hemoglobin, confounding PA quantification at varying SO_2 percentages and ICG concentrations.

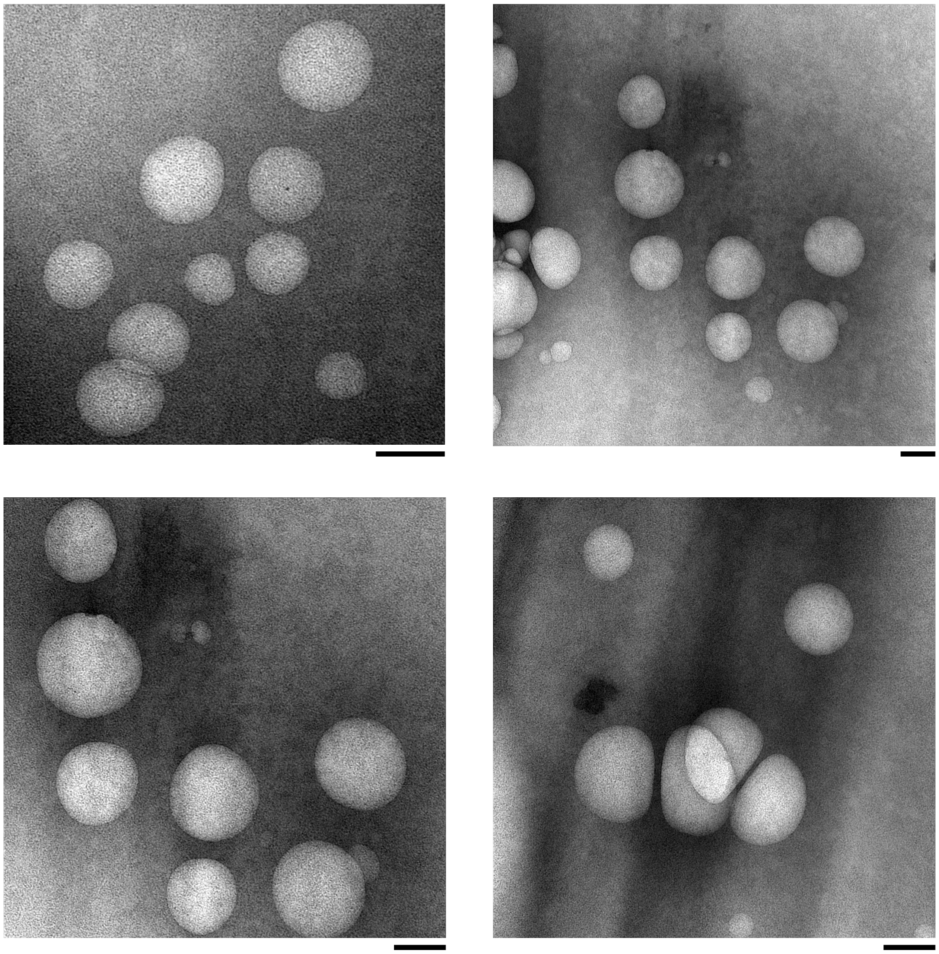

Supplementary Fig. 2: TEM images. Sample TEM images of PAtrace nanoparticles with different magnifications. Five TEM images of the same sample were used for size analysis. Black scale bar indicates 100 nm.

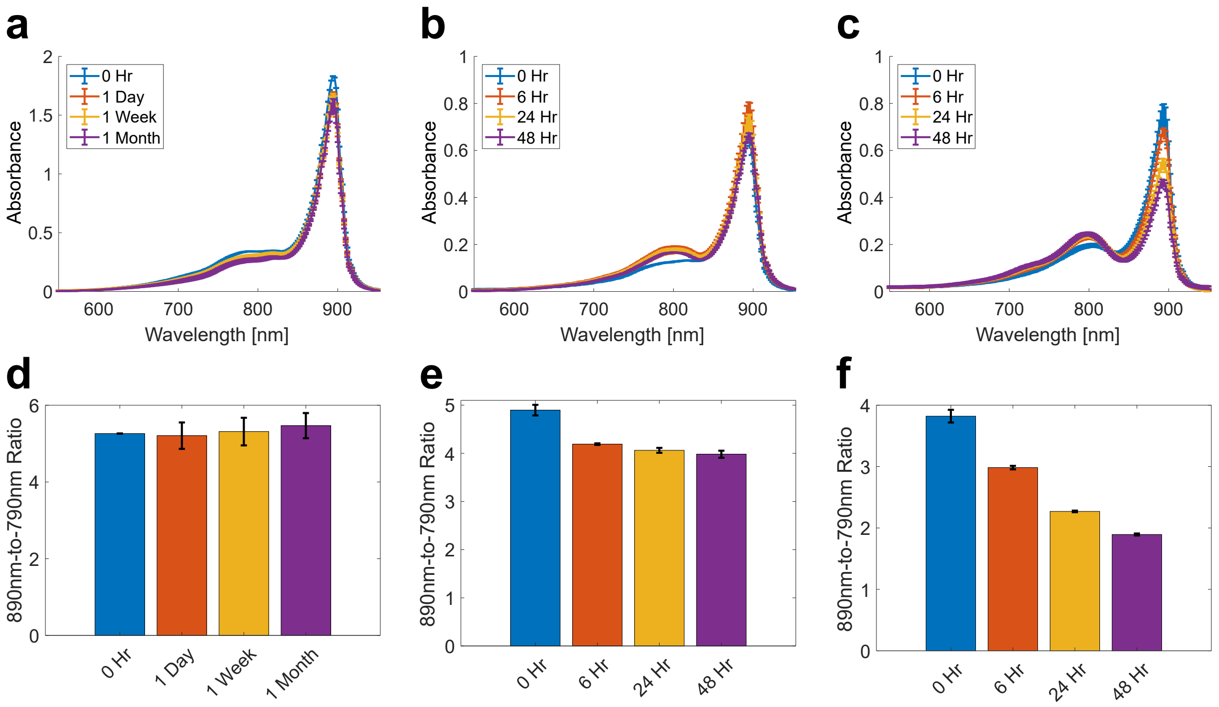

Supplementary Fig. 3: Temporal stability of PAtrace in different media. Assessment of PAtrace at (**a,d**) 4°C in PBS demonstrates minimal spectral changes over one month, indicating a long shelf life. At (**b,e**) 37°C in 10% FBS, no significant changes were observed; however, at (**c,f**) 37°C in 100% FBS, the 890nm-to-790nm ratio reduced by ~50% after 48 hr. Error bars indicate mean ± SD across three absorbance acquisitions.

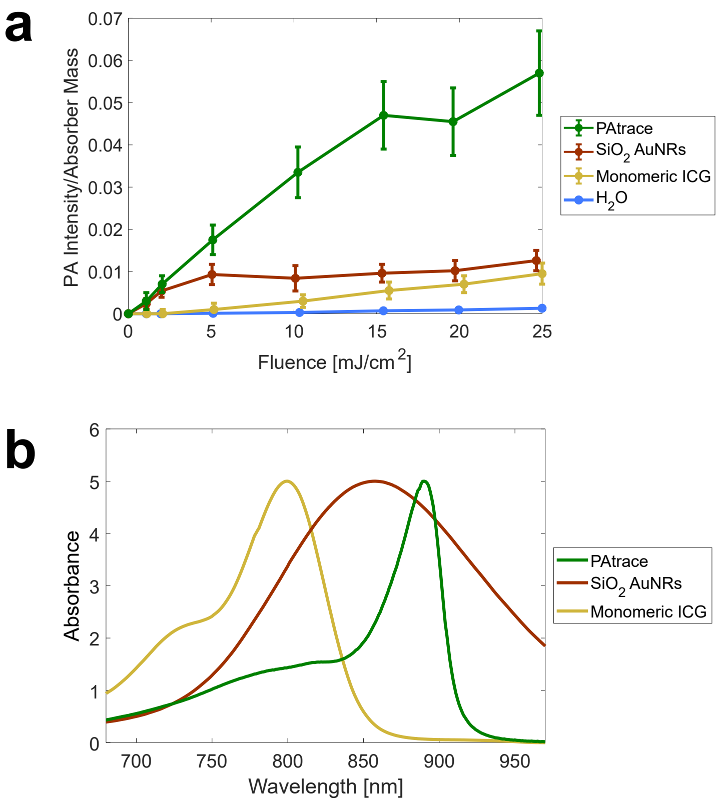

Supplementary Fig. 4: Fluence stability of imaging contrast. (**a**) PA imaging at each absorber's peak wavelength with increasing laser fluence of PAtrace (green) demonstrates high fluence stability, presenting a near-linear mass-normalized PA signal up to 15 mJ/cm^2. Conversely, SiO_2 AuNRs (maroon) become nonlinear at >2 mJ/cm^2. Error bars indicate mean ± SD across 900 laser pulses. (**b**) Corresponding optical absorbance spectra indicate a matched peak OD that was used for all particles tested. The concentration of the absorbers, with the exception of water, were controlled so that the absorbance at the peak wavelength was 5 OD.

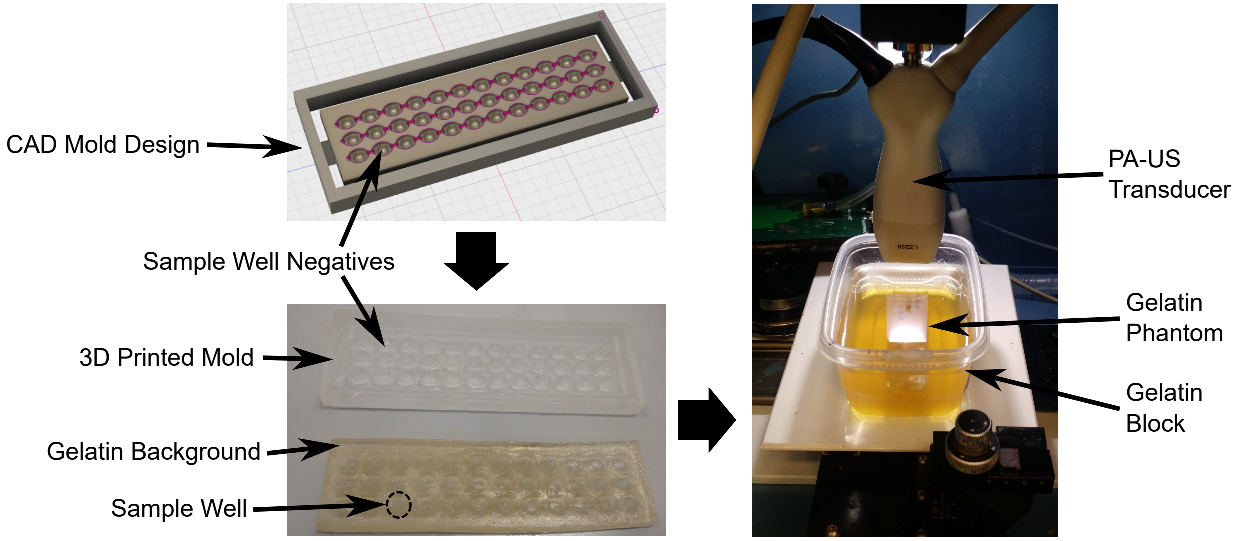

Supplementary Fig. 5: Multi-well gelatin phantom. A multi-well phantom mold was designed in CAD software (upper left) and 3D printed (lower left). The mold was used to create a gelatin phantom with 36 semi-ellipsoidal wells (lower left), and samples were decanted into each well. The phantom was adhered atop a gelatin block before imaging with the Vevo 2100 LAZR system (right).

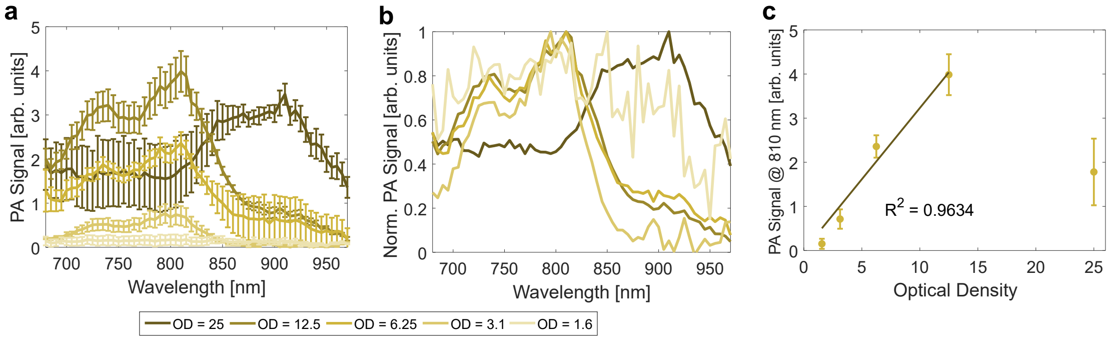

Supplementary Fig. 6: PA imaging of serial dilutions of ICG. (**a**) PA imaging of serial dilutions (25-1.6 OD) of monomeric ICG dissolved in 4% bovine serum albumin in water. Error bars indicate mean ± SD across three independent PA acquisitions. (**b**) As shown by normalized spectra, PA signal from ICG is stable up to 12.5 OD. Note that because no substantial ICG PA spectrum was observed at 1.6 OD, its normalized PA spectrum at this concentration amplifies the noise. (**c**) Peak PA signal for ICG remains linear up to 12.5 OD. Error bars indicate mean ± SD across three independent PA acquisitions.

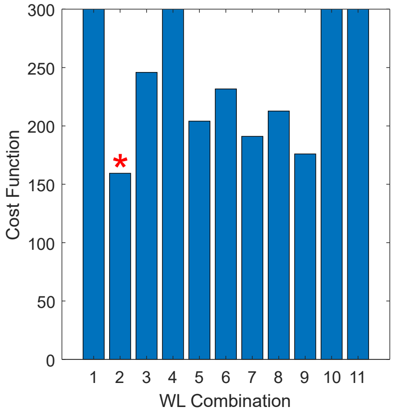

WL Combination

1	710, 734, 760, 800, 830 nm
2	710, 734, 760, 800, 830, 870 nm
3	710, 734, 760, 800, 830, 870, 890 nm
4	710, 734, 760, 800, 830, 870, 890, 900 nm
5	710, 734, 760, 800, 830, 870, 890, 900, 920 nm
6	734, 760, 800, 830, 870, 890, 900 nm
7	734, 760, 800, 830, 870, 890, 900, 920 nm
8	760, 800, 830, 870, 890, 900 nm
9	760, 800, 830, 870, 890, 900, 920 nm
10	800, 830, 870, 890, 900 nm
11	800, 830, 870, 890, 900, 920 nm

$$\text{Cost function} = \sum_{\text{ICG, PAtrace}} \left(\text{SO}_2 \text{ error} \times \text{Probe error} \times \sqrt{\text{nWL}}\right),$$

$$\text{where SO}_2 \text{ error} = \frac{1}{\text{n}_{\text{SO}_2}} \sum_{\text{n}_{\text{SO}_2}} \left|\text{SO}_2 \text{ estimate}_{\text{probe}} - \text{SO}_2 \text{ estimate}_{\text{no probe}}\right|,$$

$$\text{and Probe error} = \left|3 - \frac{\text{Probe signal}_{0.9\text{OD}}}{\text{Probe signal}_{0.3\text{OD}}}\right|.$$

Supplementary Fig. 7: Cost function for phantom wavelength selection. Optimized wavelengths for unmixing of hemoglobin and PAtrace (or ICG) were determined by a cost function (first equation, bottom; bar plot, left), which included: mean absolute SO_2 estimation error, which is the average difference between SO_2 estimates with and without probe, within the PE-tube ROI (second equation, bottom; this error was calculated and summed for the 50% & 70% SO_2 samples [i.e., $n_{SO_2} = 2$] mixed with 0.9 OD probe); absolute error in the probe signal ratio relative to the expected ratio of 3, where Probe signal is PAtrace (or ICG) signal within the ROI at the concentration denoted by the subscript (last equation, bottom); and the number of wavelengths (nWL), which accounts for the overall scan time. SO_2 and Probe error are weighted more heavily than the number of wavelengths, and the cumulative cost function is the summation of the individual ICG and PAtrace cost-function calculations to choose an optimized wavelength combination for both probes. The wavelength combination obtained from the minimum of the cost function is indicated by a red asterisk (bar graph, top left). Eleven wavelength combinations were tested (table, right).

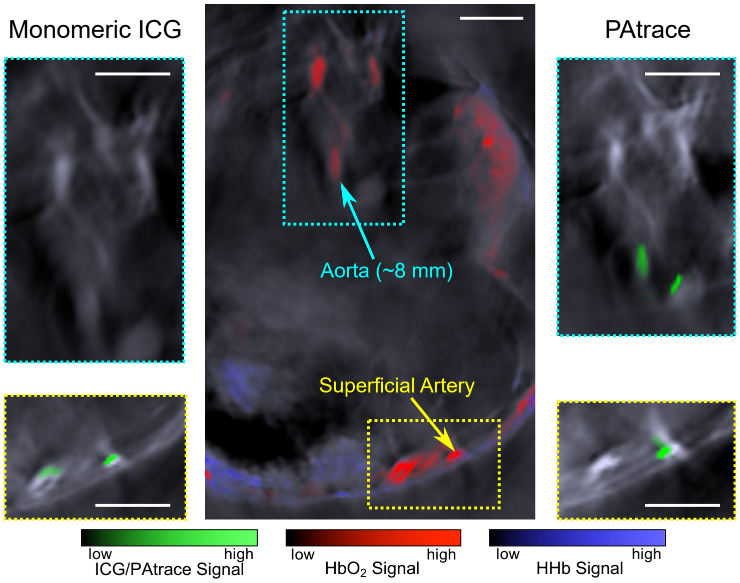

Supplementary Fig. 8: *In vivo* PA imaging comparison of ICG and PAtrace with preclinical system. Comparison of PAtrace and monomeric ICG in a wild-type mouse with the inVision preclinical PA imaging system. Each was injected intravenously at the matched ICG concentration of 0.4 mM and identified in vasculature, both superficially in an artery (yellow box) and at depth in the aorta (cyan box). Superficially (bottom panels), both monomeric ICG (left) and PAtrace (right) are easily identifiable; however, at depth (top panels), PAtrace is easily resolvable, while monomeric ICG is not. This experiment was conducted once. White scale bars indicate 3 mm.

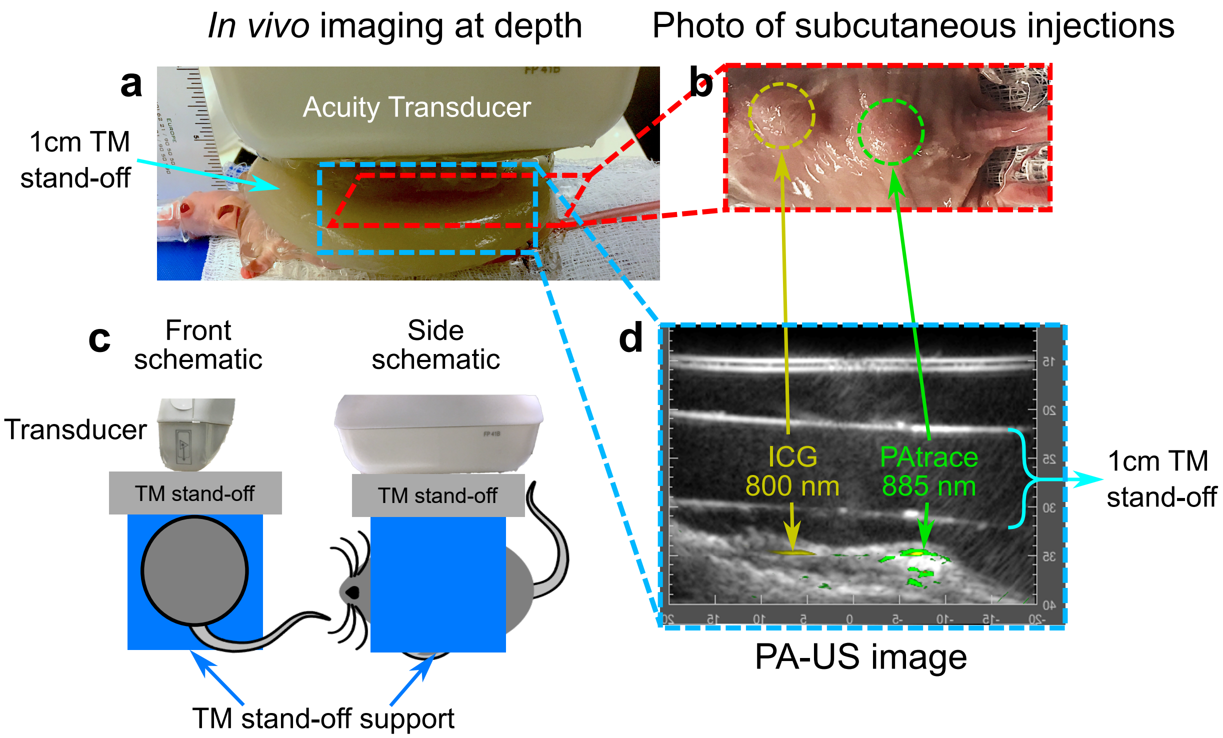

Supplementary Fig. 9: *In vivo* PA imaging comparison of ICG and PAtrace with clinical system. Comparison of PAtrace and monomeric ICG in a wild-type mouse with the Acuity clinical PA-US imaging system. (**a,c**) To image at depth, a 1cm tissue-mimicking (TM) standoff was placed between the mouse and the transducer. (**b**) Monomeric ICG and PAtrace were injected subcutaneously at the matched ICG dye concentration of 0.01 mM and imaged with the Acuity system; (**d**) each absorber was visualized at its peak wavelength. PAtrace (green) is easily resolvable at depth, whereas monomeric ICG (yellow) is much more difficult to distinguish. This experiment was conducted once. Scales for image axes provided in millimeters.

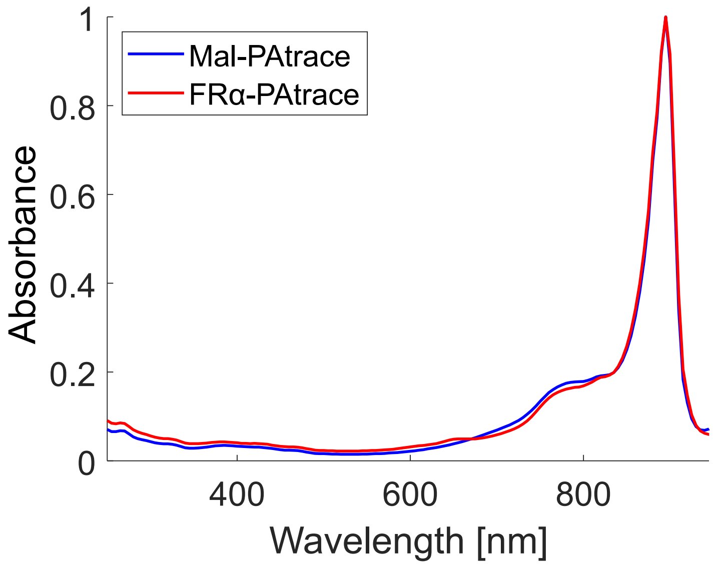

Supplementary Fig. 10: UV-Vis-NIR spectrum of antibody-conjugated vs. non-conjugated PAtrace. FRα-PAtrace (red) has the same UV-Vis-NIR spectrum as non-conjugated Mal-PAtrace (blue), demonstrating that the antibody conjugation does not affect the absorbance of the PAtrace nanoparticles.

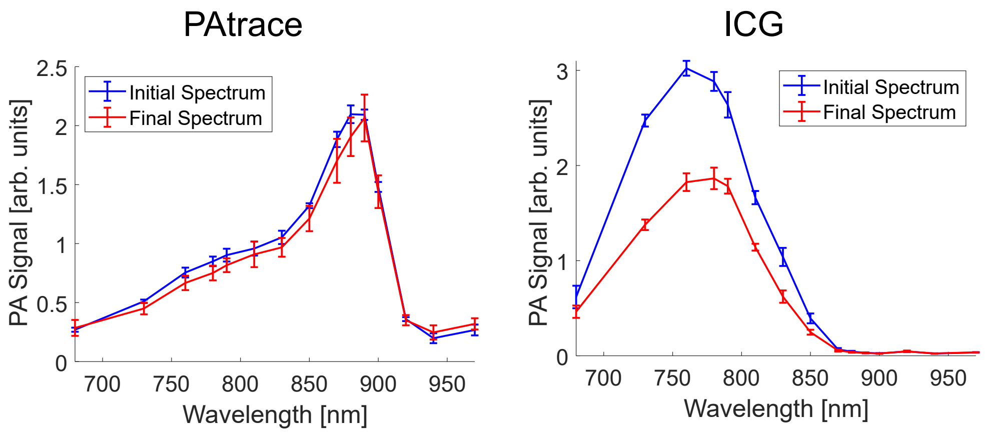

Supplementary Fig. 11: Photothermal stability of PAtrace vs. ICG. PA spectra of FRα-PAtrace (left) in PBS and monomeric ICG (right) in 50% EtOH from first irradiation spectral sequence (360 total laser pulses) and final irradiation sequence (1,440 total laser pulses) demonstrates that FRα-PAtrace particles provide greater photothermal stability than monomeric ICG. Correlation coefficients between first and last spectra are 0.99 for both FRα-PAtrace and monomeric ICG, indicating high agreement between spectral features for both. Error bars indicate mean ± SD across four independent PA acquisitions.

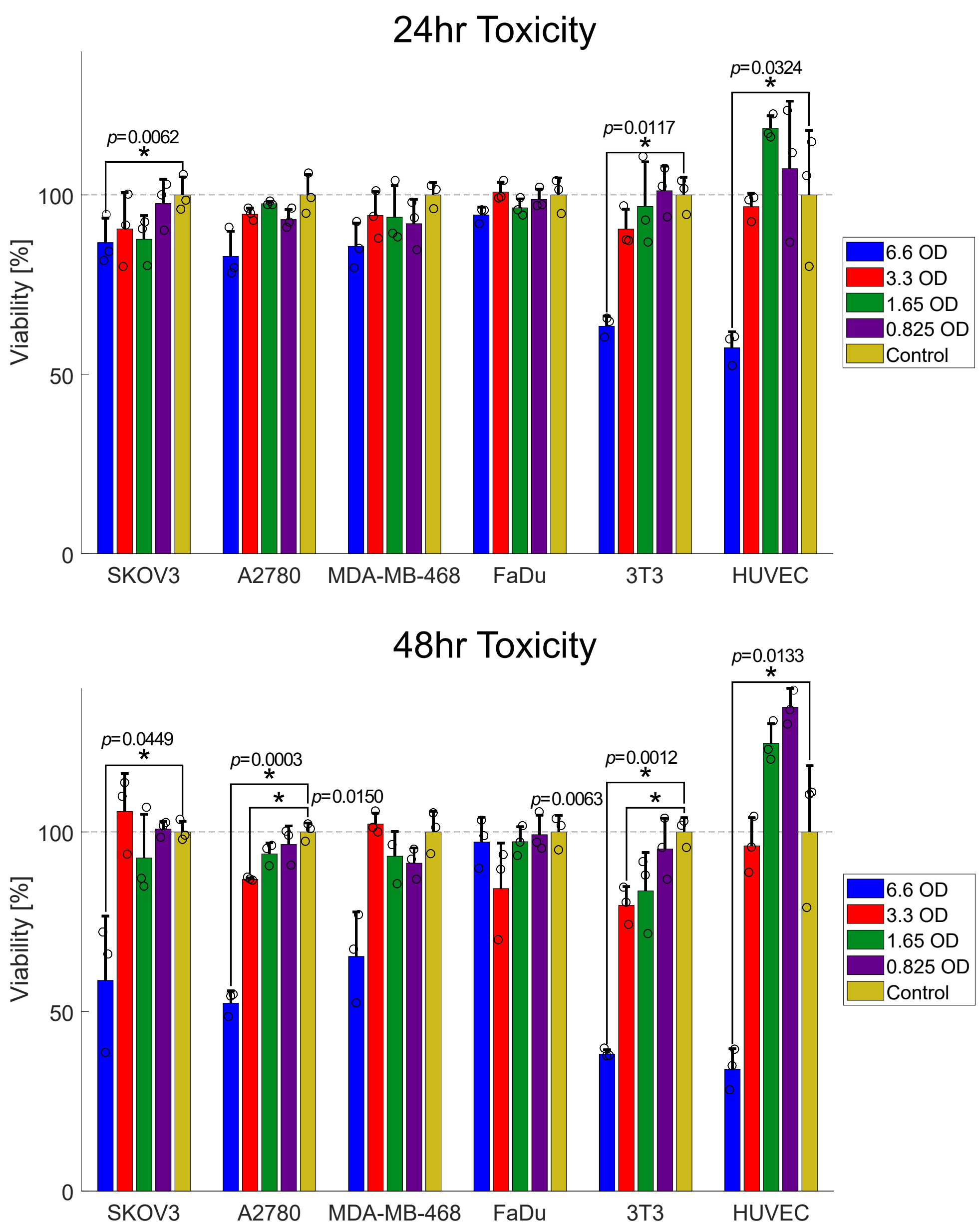

Supplementary Fig. 12: *In vitro* toxicity of PAtrace. Four cancer cell lines, 3T3 fibroblasts, and HUVEC endothelial cells were incubated with PEGylated PAtrace at varying concentrations for 24 hr (top) or 48 hr (bottom). A two-sided Student's t-test was conducted against the control, with p-values provided for significant differences. Error bars indicate mean ± SD across three independent samples.

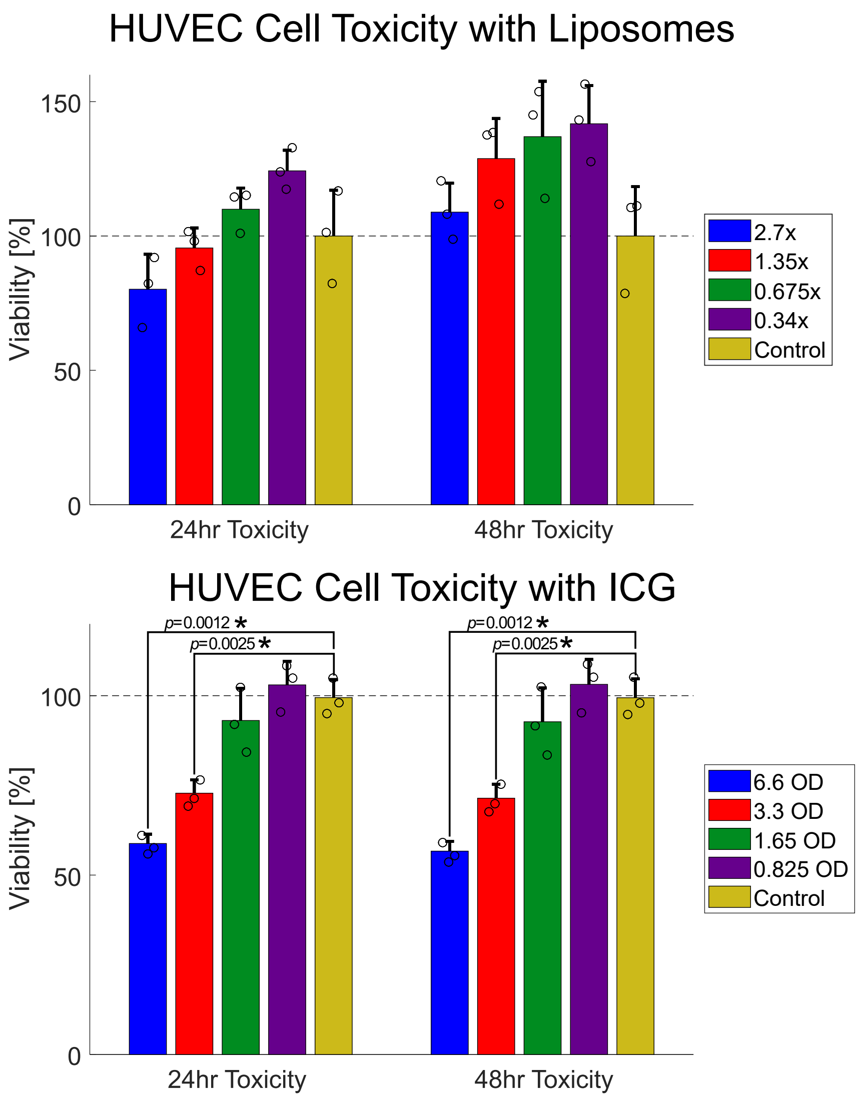

Supplementary Fig. 13: *In vitro* toxicity of empty liposomes and ICG. HUVEC endothelial cells were incubated with empty liposomes (top) or monomeric ICG (bottom) at varying concentrations for 24 hr or 48 hr. The concentration of liposomes was varied from 0.34x to 2.7x the maximum concentration of PAtrace shown in Supplementary Fig. 12. A two-sided Student's t-test was conducted against the control, with p-values provided for significant differences. Error bars indicate mean ± SD across three independent samples.

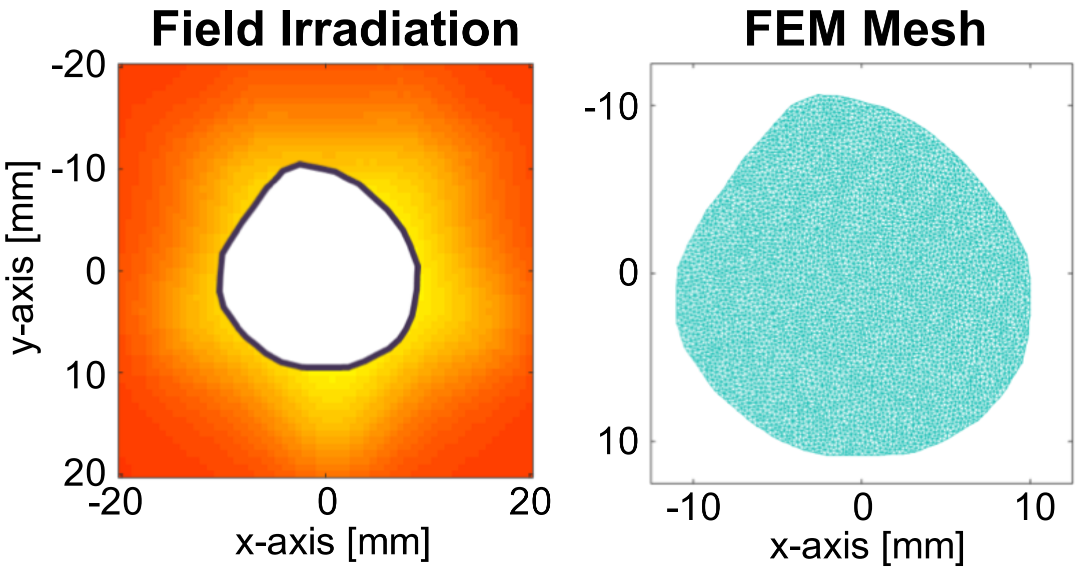

Supplementary Fig. 14: inVision field model. Example of a modeled inVision irradiation field based on the system's ring geometry (left). The mouse surface is manually segmented (black line, left), and a finite element method (FEM) mesh (right) is generated for the tissue.

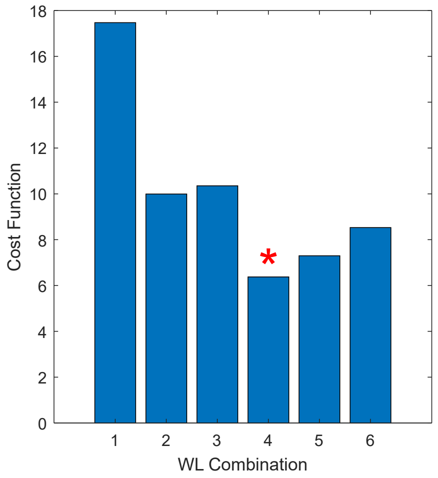

WL Combination

1	730, 760, 800, 830 nm
2	730, 760, 800, 830, 890 nm
3	730, 760, 780, 800, 830, 890 nm
4	730, 760, 780, 800, 830, 890, 910 nm
5	760, 800, 830, 890, 910 nm
6	760, 780, 800, 830, 890, 910 nm

$$\text{Cost function} = \sum_{\text{FR}\alpha,\ \text{RG-16}} \left(\Delta \text{SO}_2 \times \text{PAtrace signal}_{\text{preinjection}} \times \sqrt{\text{nWL}}\right),$$

$$\text{where } \Delta \text{SO}_2 = \left|\text{SO}_2 \text{ estimate}_{\text{postinjection}} - \text{SO}_2 \text{ estimate}_{\text{preinjection}}\right|.$$

Supplementary Fig. 15: Cost function for *in vivo* wavelength selection. Optimized wavelengths for unmixing of hemoglobin and PAtrace were determined by a cost function (first equation, bottom; bar plot, left), which included: absolute change in SO_2 estimates from preinjection to postinjection, each within a superficial-liver ROI (second equation, bottom); preinjection PAtrace signal within the ROI; and the number of wavelengths (nWL), which accounts for the overall scan time. The change in SO_2 and preinjection PAtrace signal are weighted more heavily than the number of wavelengths, and the cumulative cost function is the summation of the individual cost-function calculations for one FRα mouse and one RG-16 mouse to choose an optimized wavelength combination for both probe types. The wavelength combination obtained from the minimum of the cost function is indicated by a red asterisk. Six wavelength combinations were tested (table, right).

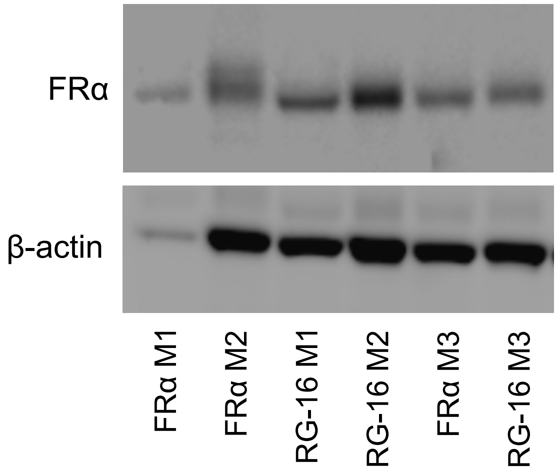

Supplementary Fig. 16: Western blot of SKOV3 tumors. FRα expression was assessed for each SKOV3 tumor with western blotting of the anti-FRα monoclonal antibody (45 kDa; top). β-actin (42 kDa; bottom) was used as a loading control and was run on the same gel as the FRα samples.

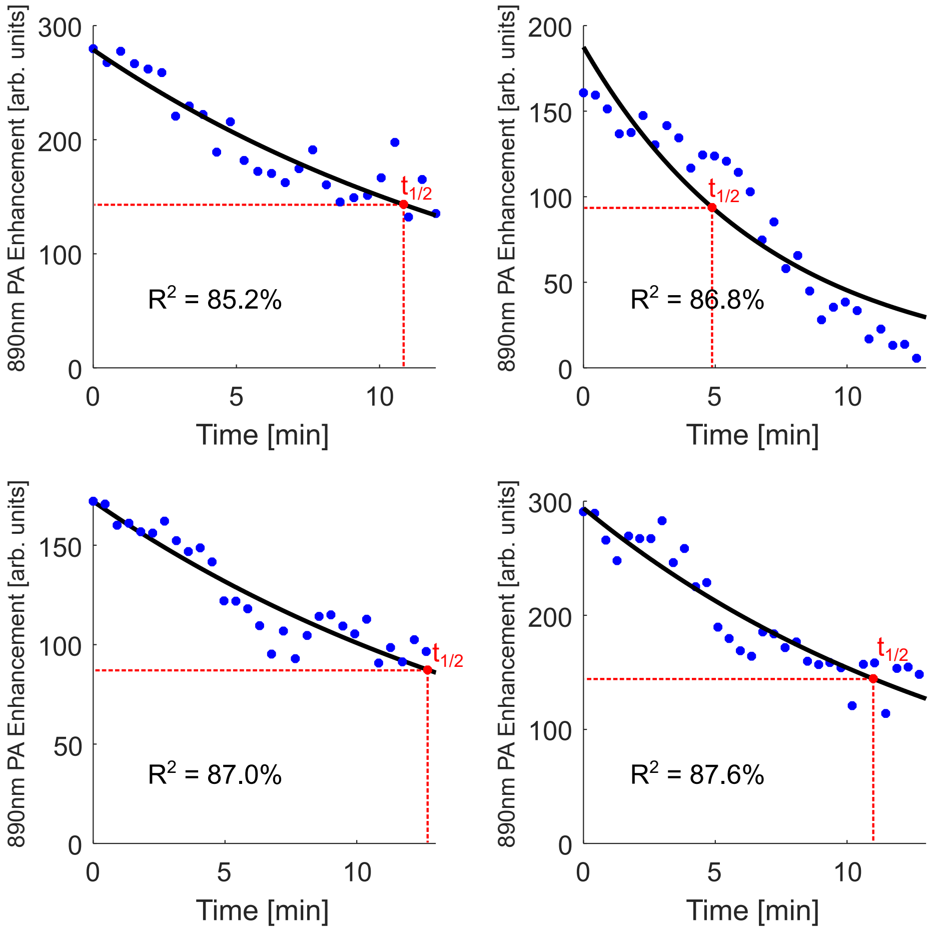

Supplementary Fig. 17: Circulation half-life of FRα-PAtrace. Circulation half-life plots from the four mice injected with FRα-PAtrace and imaged continuously for ~15 min postinjection. 890nm data were subtracted from the preinjection baseline to determine enhancement, and an ROI was selected in a superficial surface vein in the neck. Each mouse was fit to an exponential, yielding an average (±SD) circulation half-life estimate (i.e., $t_{1/2}$) of 10.0 (±3.5) min.

Supplementary Table 1: Hematology. Hematology results from five mice injected with FRα-PAtrace and one mouse that was not injected (control). Blood was harvested 24 hr postinjection. The hematology values for the FRα-PAtrace group follow closely with the control, and no significant changes are noted between the control and FRα-PAtrace groups. According to evaluation by the board-certified veterinarian, Dr. Jody L. Swain, there was slight hemolysis seen in several of the FRα-PAtrace samples, which accounts for the modest increase in platelet counts.

	**Mouse 1**	**Mouse 2**	**Mouse 3**	**Mouse 4**	**Mouse 5**	**Control**
**WBC (10^3/µL)**	3.36	3.6	2.93	5.17	3.15	4.22
**RBC (10^6/µL)**	9.47	9.21	8.85	10.11	9.26	7.78
**Hgb (g/dL)**	14.8	14.8	14.7	16.2	14.7	12.4
**Hct (%)**	46.7	45.9	45	48	45.9	38.9
**MCV (fL)**	49.3	49.9	50.8	47.5	49.6	50.1
**MCH (pg)**	15.7	16	16.6	16.1	15.9	15.9
**MCHC (g/dL)**	31.8	32.1	32.6	33.8	32.1	31.7
**Platelets (10^3/µL)**	1413	1290	1309	1413	1289	790
**Neut (10^3/µL)**	1.28	2.34	1.14	3.35	1.51	1.76
**Lymph (10^3/µL)**	1.68	0.92	1.61	1.49	1.41	1.53
**Mono (10^3/µL)**	0.2	0.08	0.06	0.14	0.04	0.18
**Neut (%)**	38	65	39	64.8	48	41.8
**Lymph (%)**	50	25.6	55	28.9	44.8	36.4
**Mono (%)**	6	2.2	2	2.7	1.4	4.3

Supplementary Table 2: Blood chemistry. Blood chemistry results from five mice injected with FRα-PAtrace and one mouse that was not injected (control). Blood was harvested 24 hr postinjection. The blood chemistry values for the FRα-PAtrace group follow closely with the control, and no significant changes are noted between the control and FRα-PAtrace groups. According to evaluation by the board-certified veterinarian, Dr. Jody L. Swain, there was slight hemolysis seen in several of the FRα-PAtrace samples, which accounts for the modest increases in AST and LDH values.

	**Mouse 1**	**Mouse 2**	**Mouse 3**	**Mouse 4**	**Mouse 5**	**Control**
**Albumin (g/dL)**	3.41	3.15	3.25	3.15	3.23	2.54
**ALP (U/L)**	61	53	75	61	68	55
**ALT (U/L)**	29	50	34	42	29	24
**AST (U/L)**	113	172	83	162	138	96
**BUN (mg/dL)**	19.8	27	23.8	34	28.7	26.5
**Calcium (mg/dL)**	9.8	10.4	10	9.8	10.8	9.9
**Chloride (meq/L)**	110.6	113.8	113.1	114.9	109.5	113.8
**Creatinine (mg/dL)**	0.24	0.21	<0.2	<0.2	<0.2	<0.2
**Globulin (g/dL)**	1.19	1.01	0.99	1.17	1.19	1.55
**Potassium (meq/L)**	4.53	3.34	3.49	3.62	4.67	4.5
**LDH (U/L)**	794	900	165	1639	336	346
**Sodium (meq/L)**	149.8	154.4	151.1	156.8	151.4	148.5
**Phosphorus (mg/dL)**	6	7.6	6.6	6.6	8.8	6.1
**Glucose (mg/dL)**	128	184	279	116	144	148
**Total Protein (g/dL)**	4.6	4.15	4.24	4.32	4.42	4.08

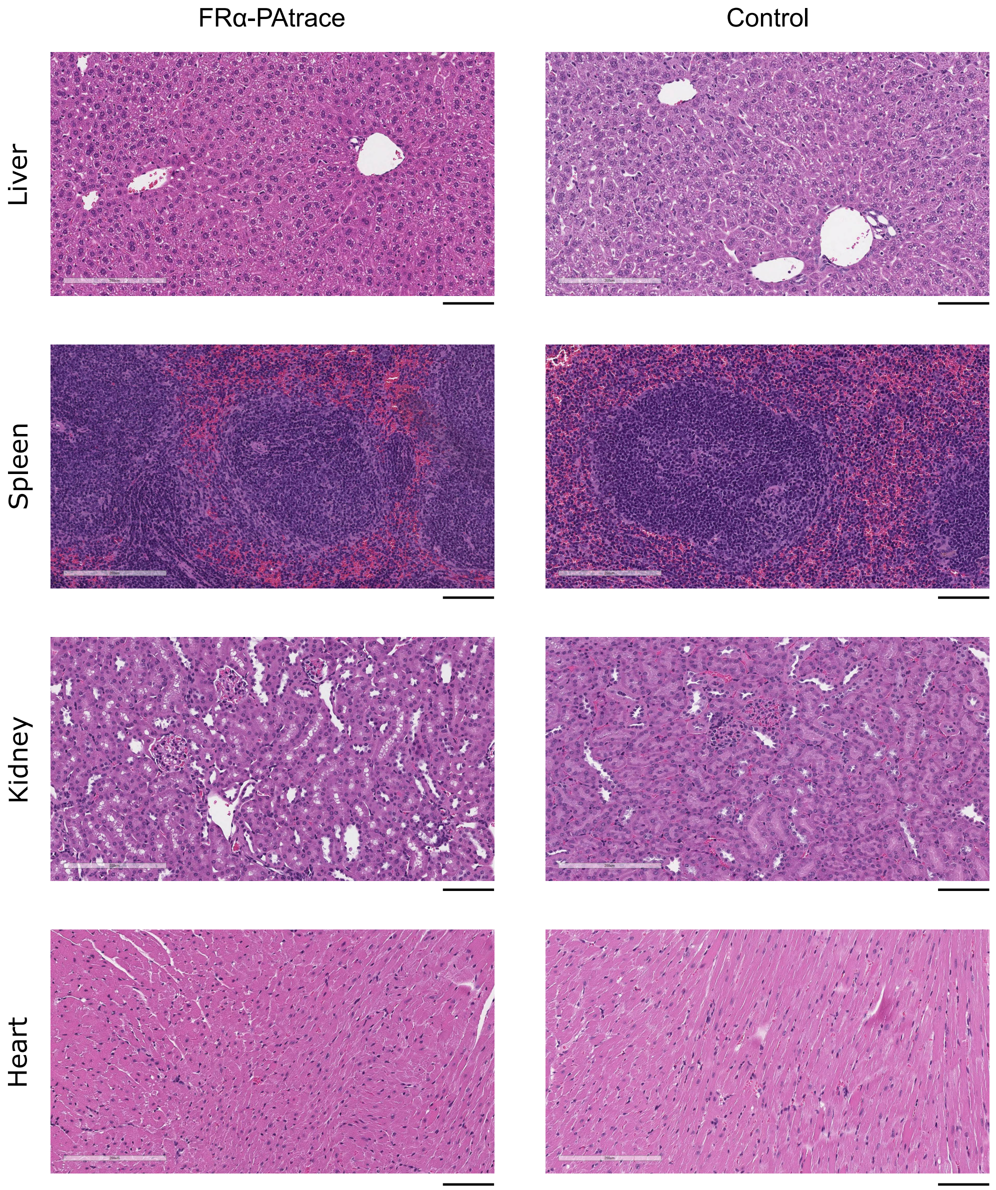

Supplementary Fig. 18: Representative histology images. Samples of liver, spleen, kidney, and heart were harvested from five mice injected with FRα-PAtrace (left) and one control mouse that was not injected (right). Samples were formalin-fixed, paraffin-embedded, stained with H&E, and examined microscopically by a board-certified veterinary pathologist, Dr. Elizabeth Whitley. There were no observable morphologic changes related to the FRα-PAtrace injection in any of the samples. Black scale bar to the bottom-right of each image indicates 100 µm.
