# Supplementary material for: Clinically translatable quantitative molecular photoacoustic imaging with liposome-encapsulated ICG J-aggregates
Source: Nat Commun. 2021 Sep 13;12:5410. doi: 10.1038/s41467-021-25452-3 (PMC8438038; doi:10.1038/s41467-021-25452-3)
Supplement: Supplementary file 3 — Reporting Summary [file 41467_2021_25452_MOESM3_ESM.pdf]

nature portfolio

Corresponding author(s): Richard Bouchard, Konstantin Sokolov

Last updated by author(s): Jun 24, 2021

Reporting Summary

Nature Portfolio wishes to improve the reproducibility of the work that we publish. This form provides structure for consistency and transparency in reporting. For further information on Nature Portfolio policies, see our Editorial Policies and the Editorial Policy Checklist.

Statistics

For all statistical analyses, confirm that the following items are present in the figure legend, table legend, main text, or Methods section.

n/a	Confirmed	
☐	☒	The exact sample size (n) for each experimental group/condition, given as a discrete number and unit of measurement
☐	☒	A statement on whether measurements were taken from distinct samples or whether the same sample was measured repeatedly
☐	☒	The statistical test(s) used AND whether they are one- or two-sided<br>*Only common tests should be described solely by name; describe more complex techniques in the Methods section.*
☐	☒	A description of all covariates tested
☒	☐	A description of any assumptions or corrections, such as tests of normality and adjustment for multiple comparisons
☐	☒	A full description of the statistical parameters including central tendency (e.g. means) or other basic estimates (e.g. regression coefficient) AND variation (e.g. standard deviation) or associated estimates of uncertainty (e.g. confidence intervals)
☐	☒	For null hypothesis testing, the test statistic (e.g. F, t, r) with confidence intervals, effect sizes, degrees of freedom and P value noted<br>*Give P values as exact values whenever suitable.*
☒	☐	For Bayesian analysis, information on the choice of priors and Markov chain Monte Carlo settings
☒	☐	For hierarchical and complex designs, identification of the appropriate level for tests and full reporting of outcomes
☒	☐	Estimates of effect sizes (e.g. Cohen's d, Pearson's r), indicating how they were calculated

Our web collection on statistics for biologists contains articles on many of the points above.

Software and code

Policy information about availability of computer code

Data collection	Gen5; Fusion 360; ViewMSOT 3.8
Data analysis	ImageJ 3.0; Vevo LAB 3.2; MATLAB R2019b; 3DSlicer 4.10; NIRFAST 9.1

For manuscripts utilizing custom algorithms or software that are central to the research but not yet described in published literature, software must be made available to editors and reviewers. We strongly encourage code deposition in a community repository (e.g. GitHub). See the Nature Portfolio guidelines for submitting code & software for further information.

Data

Policy information about availability of data

All manuscripts must include a data availability statement. This statement should provide the following information, where applicable:

- Accession codes, unique identifiers, or web links for publicly available datasets
- A description of any restrictions on data availability
- For clinical datasets or third party data, please ensure that the statement adheres to our policy

All data supporting the findings provided in the manuscript and the supplementary information are available from the corresponding authors on reasonable request. Source data are provided with this paper. TEM images are provided at: https://www.ebi.ac.uk/pdbe/emdb/empiar/deposition/id/1237/open_metadata/.

Field-specific reporting

Please select the one below that is the best fit for your research. If you are not sure, read the appropriate sections before making your selection.

- [x] Life sciences
- [ ] Behavioural & social sciences
- [ ] Ecological, evolutionary & environmental sciences

For a reference copy of the document with all sections, see nature.com/documents/nr-reporting-summary-flat.pdf

Life sciences study design

All studies must disclose on these points even when the disclosure is negative.

Sample size	For the pilot in vivo photoacoustic imaging study and in vitro assays, the sample size per group (N=3) was selected as the minimum where we could reasonably obtain estimates of the mean and variance within each group. The sample size (N=5) for blood chemistry and hematology assays was increased due to the potential for increased variability in some of these metrics.
Data exclusions	No data were excluded.
Replication	Multiple PAtrace preparations were used throughout this study. Each preparation was characterized by UV-Vis-NIR spectrophotometry. Each antibody-conjugated PAtrace sample was evaluated for folate receptor binding specificity. All in vitro assays were carried out at least in triplicate. All in vitro attempts at replication were successful. No attempts were made to replicate in vivo experiments.
Randomization	All samples were randomly assigned to different wells for photoacoustic imaging in multi-well phantoms. This randomization diminishes bias due to potential variations in fluence distribution across the phantom. For the in vivo photoacoustic imaging study, mice from the same litter were surgically inoculated with identical tumors on the same day. On the day of imaging, each mouse was randomly assigned for injection with either targeted or non-targeted PAtrace nanoparticles.
Blinding	Histological organ evaluation of mice injected with PAtrace vs. controls were blinded (i.e., an evaluating veterinary pathologist was not informed of mouse assignments prior to evaluation). As evaluation of blood assays was reported relative to control-mouse values, which are specific to the particular mouse strain used, it was not blinded to the evaluating veterinarian. In vivo photoacoustic imaging and data analysis were carried out by the same investigators, so this study was not blinded. In vitro experiments and data analysis were carried out by the same investigator, so these studies were not blinded.

Reporting for specific materials, systems and methods

We require information from authors about some types of materials, experimental systems and methods used in many studies. Here, indicate whether each material, system or method listed is relevant to your study. If you are not sure if a list item applies to your research, read the appropriate section before selecting a response.

Materials & experimental systems

n/a	Involved in the study	
[ ]	[x]	Antibodies
[ ]	[x]	Eukaryotic cell lines
[x]	[ ]	Palaeontology and archaeology
[ ]	[x]	Animals and other organisms
[x]	[ ]	Human research participants
[x]	[ ]	Clinical data
[x]	[ ]	Dual use research of concern

Methods

n/a	Involved in the study	
[x]	[ ]	ChIP-seq
[x]	[ ]	Flow cytometry
[x]	[ ]	MRI-based neuroimaging

Antibodies

Antibodies used	Anti-RG-16 monoclonal antibodies (clone RG-16, Sigma-Aldrich Cat# I0138, RRID:AB_260099).<br>Anti-FRα monoclonal antibodies (clone 548908, Thermo Fisher Scientific Cat# MA5-23917, RRID:AB_2609390).<br>Anti-β-actin monoclonal antibodies (clone AC-15, Sigma-Aldrich Cat# A5441, RRID:AB_476744).<br>HRP-conjugated anti-mouse secondary IgG antibodies (affinity purified, Thermo Fisher Scientific Cat# 45-000-679).
Validation	Anti-RG-16 monoclonal antibody<br>The manufacturer provided antibody validation by ELISA. 1:40,000 Monoclonal Anti-Rabbit Immunoglobulins (IgG, IgA, IgM) antibody produced in Mouse, Clone: RG-16 (Cat. No. I0138) followed by 1:500 Anti-Mouse IgG (Fab specific)-Peroxidase antibody produced in Goat (Cat. No. A9917), was probed to detect Rabbit Immunoglobulins by indirect ELISA. Reactivity is observed with Rabbit IgG, Rabbit IgA and Rabbit IgM but not with Human IgG, Human IgA and Human IgM.<br><br>Anti-FRα monoclonal antibody<br>The manufacturer provided antibody validation by western blot and immunocytochemical analysis. Western blot analysis from lysates of human cortex tissue and HeLa human cervical epithelial carcinoma cell line. PVDF membrane was probed with 2 μg/mL of

mouse Anti-human FOLR1 Monoclonal Antibody (Product # MA5-23917) followed blotting with HRP-conjugated Anti-mouse IgG Secondary Antibody (# (SQ) 45000679 ECL™ Anti-Mouse IgG, Horseradish Peroxidase). A specific band was detected for FOLR1 at approximately 40 kDa (as indicated). This experiment was conducted using NuPAGE® Novex® 4-12% Bis-Tris Protein Gels. Immunocytochemical analysis of FOLR1 was detected in immersion fixed MCF-7 human breast cancer cell line using 10 µg/mL mouse Anti-human FOLR1 Monoclonal Antibody (Product # MA5-23917) for 3 hours at room temperature.

Anti-β-actin antibody
The manufacturer provided antibody validation by immunoblotting. Cell line lysates were separated on SDS-PAGE and probed with 1:5,000 Monoclonal Anti-β-Actin Clone: AC-15 (Cat. No. A5441). The antibody was developed using Goat Anti-Mouse IgG-Peroxidase (Cat. No. A2304) and a chemiluminescent substrate.

Eukaryotic cell lines

Policy information about cell lines

Cell line source(s)	MDA-MB-468 (RRID:CVCL_0419), NIH 3T3 (RRID:CVCL_0594), FaDu (RRID:CVCL_1218), A2780 (RRID:CVCL_0134), SKOV3 (RRID:CVCL_0532), HUVEC (RRID:CVCL_2959). HUVEC cells were received from Thermo Fisher Scientific, while all other cells were received from American Type Culture Collection.
Authentication	All cell lines were authenticated by the supplier. In addition, SKOV3 and A2780 cells were authenticated by the short tandem repeat method using the Power Plex 16HS kit (Promega). No additional authentication was used for MDA-MB-468, NIH 3T3, FaDu, A2780, SKOV3, or HUVEC cells.
Mycoplasma contamination	All cell lines were tested negative for mycoplasma by suppliers. In addition, SKOV3 and A2780 cells were tested negative using Universal Mycoplasma Detection Kit (ATCC 30-1012K).
Commonly misidentified lines (See ICLAC register)	No commonly misidentified cell lines were used in the study.

Animals and other organisms

Policy information about studies involving animals; ARRIVE guidelines recommended for reporting animal research

Laboratory animals	Athymic nu/nu mice, female, 6 weeks. Housing conditions were maintained as follows: light cycles were 12hr on (7a.m. - 7p.m.) and 12hrs off (7p.m. - 7a.m.); temperature was maintained at 72 Fahrenheit with 45% humidity.
Wild animals	No wild animals were used.
Field-collected samples	No field-collected samples were used.
Ethics oversight	Institutional Animal Care and Use Committee of the MD Anderson Cancer Center

Note that full information on the approval of the study protocol must also be provided in the manuscript.
